# The role of muscle disuse in muscular and cardiovascular fitness: A systematic review and meta‐regression

**DOI:** 10.1002/ejsc.12093

**Published:** 2024-03-18

**Authors:** Rafael A. Casuso, Jesús R. Huertas, Jerónimo Aragón‐Vela

**Affiliations:** ^1^ Department of Health Sciences Universidad Loyola Andalucía Córdoba Spain; ^2^ Department of Physiology Institute of Nutrition and Food Technology University of Granada Granada Spain; ^3^ Department of Health Sciences Area of Physiology University of Jaen Jaen Spain

**Keywords:** aerobic fitness, biomechanics, musculoskeletal, physiotherapy, strength

## Abstract

We aimed to assess the effects of muscle disuse on muscle strength (MS), muscle mass (MM) and cardiovascular fitness. Databases were scrutinized to identify human studies assessing the effects of muscle disuse on both (1) MM and (2) maximal oxygen uptake (VO_2max_) and/or MS. Random‐effects meta‐analysis and meta‐regression with initial physical fitness and length of the protocol as a priori determined moderators were performed. We quantitatively analyzed 51 different studies, and the level of significance was set at *p* < 0.05. Data from the participants in 14 studies showed a decline in both VO_2max_ (SMD: −0.93; 95% CI: −1.27 to −0.58) and MM (SMD: −0.34; 95% CI: −0.57 to −0.10). Data from 47 studies showed a decline in strength (−0.88; 95% CI: −1.04 to −0.73) and mass (SMD: −0.47; 95% CI: −0.58 to −0.36). MS loss was twice as high as MM loss, but differences existed between anatomical regions. Notably, meta‐regression analysis revealed that initial MS was inversely associated with MS decline. VO_2max_ and MS decline to a higher extent than MM during muscle disuse. We reported a more profound strength loss in subjects with high muscular strength. This is physiologically relevant for athletes because their required muscular strength can profoundly decline during a period of muscle disuse. It should however be noted that a period of muscle disuse can have devastating consequences in old subjects with low muscular strength.

## Highlights

- Disuse induces a greater decline in muscular and cardiovascular fitness than in muscle mass (MM).
- Initial fitness level is inversely associated with fitness decline but not with MM loss.
- Muscle strength loss is likely due to neural rather than muscular factors.
- The role of muscular factor on strength loss can change depending on the anatomical region.

## 1 INTRODUCTION

Skeletal muscle (SKM) health is a hallmark of physical and mental wellbeing (de Santana et al., 2021; Visser et al., 2005), and SKM wasting has been linked with disease and mortality risk (Fearon et al., 2012). Reductions in mechanical load, such as those occurring during hospitalization, immobilization or spaceflight, result in physiological adaptations leading to reduced muscle mass (MM) and muscle strength (MS; Clark, 2009).

SKM is a highly plastic tissue that responds rapidly to nutritional stimuli. However, leucine supplementation (7.5 g/day) does not prevent either MM or MS loss in healthy young subjects undergoing unilateral leg immobilization (Backx et al., 2018). In fact, when protein quantity and quality are manipulated during muscle disuse, muscular function shows an inconsistent response (Howard et al., 2020). It has been proposed that the lack of mechanical load is the main driver of disuse‐induced muscle atrophy (Oikawa et al., 2019). It is therefore unsurprising that the combination of physical activity and nutritional manipulation is an optimal approach to prevent the loss of muscular function when step reduction is used as a muscle disuse protocol (Nunes et al., 2022). However, physical activity and/or nutritional manipulations are not always feasible during disuse (i.e., immobilization or common hospitalization). Thus, understanding the mechanisms underlying the effects of muscle disuse is a goal to improve SKM physiological decline.

In this regard, prior research has indicated that prehabilitation proves inadequate in preventing MM loss during short‐term bed rest among older adults (Smeuninx et al., 2021). Notably, there is a suggestion that neural factors, rather than morphological factors, serve as the primary regulators of MS decline due to immobilization (Clark, 2009). Thus, evaluating MS may hold greater relevance than MM assessment when gauging physical deterioration resulting from muscle disuse. In addition, a recent meta‐regression analysis involving studies on bed rest has revealed an inverse correlation between the initial maximal oxygen uptake (VO_2max_) and the subsequent decline in VO_2max_ (Ried‐Larsen et al., 2017). Moreover, it has been reported that the initial muscle quadriceps volume (Qvol) exhibited an inverse association with Qvol decline, particularly observed in younger subjects and not as prominently in older subjects (Suetta et al., 2009).

A recent systematic review and meta‐analysis by Preobrazenski et al. (2023) quantified changes in MS and MM in the knee extensors following single‐leg immobilization. The study reported that declines in MS and MM tend to plateau after 14 days of disuse. Furthermore, a more significant reduction in MS compared to MM was observed in response to immobilization. However, these findings are specific to the knee extensors and the immobilization disuse protocol. This is important to consider because muscle fibre composition varies by anatomical region. For example, arm muscles predominantly contain type II glycolytic fibres (about 65%), whereas calf muscles are primarily composed of type I oxidative fibres (approximately 75%; Saltin et al., 1977; Zinner et al., 2016). Additionally, the impact of muscle disuse can differ between older and younger subjects. For instance, aged muscles tend to maintain a relatively higher capacity for maximal strength compared to power (Izquierdo et al., 1999). Hence, both age and anatomical region are crucial factors that can influence the degree of MS and MM loss due to muscle disuse.

However, no preceding meta‐analysis has conducted a comparative examination of the impacts of muscle disuse on both MS and MM. Furthermore, there has been a lack of analysis on the influence of factors such as age, muscle disuse protocols, anatomical regions, intervention duration, physical fitness, and muscle hypertrophy on the decline of MM and MS in response to muscle disuse. Consequently, the primary objective of this study is to comprehensively synthesize the effects of muscle disuse on both MM and MS, while also investigating the potential impact of various moderators on MM and MS. This endeavor aims to contribute to the formulation of tailored prehabilitation protocols applicable to a spectrum of individuals ranging from those with frailty to well‐trained subjects.

## 2 METHODS

### 2.1 Experimental approach to the problem

The study methodology follows the Preferred Reporting Items for Systematic Reviews and Meta‐Analyses (PRISMA) 2020 statement (Page et al., 2021) and the recently published PRISMA in Exercise, Rehabilitation, Sport medicine and SporTs science guide (Ardern et al., 2022). The protocol was registered in the International Prospective Register of Systematic Reviews database (CRD42021254605). The PubMed and Web of Science databases were systematically searched for eligible articles up to December 31, 2022. The PICO framework is as follows: population: healthy subjects, intervention: bed rest or immobilization or hindlimb unload, comparison: pre intervention versus post intervention; outcomes: VO_2max_, MS, MM. Both cohort and randomized controlled trials were included. This is because some studies uses protocols exploring just one group submitted to an atrophy protocol and other studies reported a muscle disuse protocol in combination with a countermeasure, in this case data from the control group was used. Any study on healthy human subjects (≥18 years old) with an experimental group targeting the effects of bed rest, limb immobilization or limb unloading on (1) muscle atrophy and (2) VO_2max_ or MS was considered for this meta‐analysis. Only studies published in English were included. The following search strategy was used: (“aerobic capacity” or “endurance capacity” or “VO_2max_” or “maximal aerobic power” or “VO_2peak_” or “physical fitness” or “fitness” or “MS” or “maximal voluntary contraction” [MVC] or “muscle function”) and (“MM” or “muscle volume” or “atrophy” or “muscle size” or “muscle thickness”) and (“immobilization” or “immobilization” or “limb unload” or “bed rest” or “bedrest” or “bed‐rest”). To identify missing studies, each selected study was individually scrutinized by clicking on the “cited” and “similar” tabs of the databases.

### 2.2 Study selection

The selection of the studies was independently performed by two reviewers (RAC and JAV). Studies were screened based on their titles and abstracts. In the case that a study abstract reported a muscle disuse protocol in combination with a countermeasure (i.e., exercise or nutrition intervention), the full text was assessed as the control group could meet the inclusion criteria. Discrepancies during the study selection were resolved by consensus and/or by the opinion of a third author (JRH).

### 2.3 Data extraction and quality assessment

The following data were independently extracted by two researchers (RAC and JAV): number of subjects, sex, age, body mass index, weight, height, disuse protocol, anatomical region, length of the protocol, strength protocol, MS, VO_2max_, MM, and MM protocol. Where data were not presented in the text or in tables and the authors could not be reached, data were extracted using WebPlotDigitalizer (Jelicic Kadic et al., 2016). Any discrepancies between reviewers were resolved by consensus.

We identified three articles studying different bed rest countermeasures, that is, protein supplementation (Arentson‐Lantz, Galvan, Ellison, et al., 2019), exercise (Arentson‐Lantz, Galvan, Wacher, et al., 2019) and leucine (Arentson‐Lantz et al., 2020), and sharing the same control group. Similarly, two studies analyzing the effects of leucine (Backx et al., 2018) and creatine (Backx et al., 2017) shared the control group. In these two cases, we only included the data of the shared control group. In addition, we could not extract data from two articles, as the data were reported as changes (Cook et al., 2010, 2014).

### 2.4 Data synthesis and analysis

All analyses were performed using the metafor package of R software (Viechtbauer, 2010). The meta‐analyses were performed using random‐effects models with DerSimonian‒Laird methods to assess the effect of muscle disuse on MS, VO_2max_, and MM. We calculated the effects size using hedges's *g* as previously performed by a study with a similar design (Pérez‐Rodríguez et al., 2023). Heterogeneity between studies was assessed using Cochran *Q* and *I* ^2^ statistics. Effect sizes are presented as the mean difference (MD) and 95% CIs or standardized mean difference (SMD) and 95% CIs when the outcome had noncomparable scales. All data on MS and MM were collected for each study. However, to prevent inflation of the results, only one marker was included from each study. For MM, muscle volume was chosen over cross‐sectional area (CSA) and CSA over leg lean mass. When multiple strength outcomes were collected, MVC was selected over one repetition maximum. In addition, isometric MVC was selected over isokinetic MVC based on the number of studies providing each measurement.

For subgroup analysis “old” age was considered as a mean age of >60 years and “young” as a mean age of <60 years. One study (Dirks et al., 2016) reported VO_2max_ data as mL/min; for the meta‐regression, the initial value was normalized to kg. For the studies including strength, subgroup analysis was performed for age, anatomical region (thigh, calf and arm) and protocol (bed rest, immobilization and unloading).

In addition, it has been reported that both the length of the intervention and initial physical fitness can potentially alter the response to muscle disuse (Dirks et al., 2016). Therefore, we conducted a meta‐regression for four a priori chosen moderators: (1) initial VO_2max_, (2) initial MS, (3) initial MM and (4) length of the intervention. The number of VO_2max_ studies assessing MM in comparable units was insufficient (<10); thus, only the length (days) and the initial VO_2max_ (mL/kg/min) were tested for moderators of VO_2max_ change and MM loss. For strength studies, the leg (thigh) was the only anatomical region with enough studies to include initial strength (Nm), length (days), muscle volume (mL) and muscle CSA (cm^2^) as moderators for both MS and MM loss.

### 2.5 Heterogeneity and risk of bias

We performed similar methods as previously published (Galan‐Lopez & Casuso, 2023). Heterogeneity was reported as the *I* ^2^ value and the prediction interval derived from tau. The Cochrane Handbook suggests that *I* ^2^ ranging from 75% to 100% represent a considerable heterogeneity (*Chapter 10: Analyzing Data and Undertaking Meta‐Analyses | Cochrane Training*, n.d.). Publication bias was assessed using visual inspection of funnel plots for asymmetries, in addition, Egger tests was used to quantify publication bias by analyzing funnel plot asymmetries. Publication bias pertains to the tendency of significant results being more likely to be published compared with null results. A *p*‐value less than 0.05 in the Egger test indicates publication bias. The Rosenthal fail‐safe number provides an estimation of the number of studies that would be needed to nullify the observed effect (i.e., reduce it to nonsignificance; Fragkos et al., 2017). In addition, we employed the leave‐one‐out method as a sensitivity analysis to assess whether any of the included studies is influencing the overall effect. If the leave‐one‐out test yielded positive results, we reported the effect size of the model with that particular study excluded from the analysis.

The Critical Appraisal Checklist for Case Control Studies or randomized controlled trials of the Faculty of Health and Medical Sciences at the University of Adelaide, South Australia (Moola et al., 2020), was used to evaluate bias in studies (RAC and JAV). The checklist consists of 8–13 items relating to the title, abstract, introduction, methods, results and discussion sections of articles. However, not all items were applicable in every study. Therefore, quality scores were calculated both as a total of points and as a percentage of the applicable items.

## 3 RESULTS

### 3.1 Study characteristics

The original search yielded 1046 studies, and after deduplication and screening, 197 studies were independently read and reviewed. A total of 51 studies were included for quantitative analysis (Supplementary Figure S1). Participant details and outcomes are presented in Supplementary Table S1. A total of 12 studies with 14 different samples (*n* = 143 subjects) assessed both VO_2max_ and MM changes due to disuse (Akima et al., 2005; Arentson‐Lantz, Galvan, Ellison, et al., 2019; Arentson‐Lantz, Galvan, Wacher, et al., 2019; Arentson‐Lantz et al., 2016, 2020; Coker et al., 2015; Deutz et al., 2013; Dirks et al., 2019; English et al., 2016; Kramer et al., 2017; Pišot et al., 2016; Ploutz‐Snyder et al., 2018; Režen et al., 2014; Vigelsø et al., 2015). All these studies were performed under a bed‐rest protocol except for Vigelsø et al. (2015), where a one‐leg immobilization protocol was applied. We also found 47 studies with 54 different samples (*n* = 570 subjects) assessing both MS and MM changes following muscle disuse. Twenty‐eight studies provided data from a bed‐rest protocol (Akima et al., 2000, 2005; Arc‐Chagnaud et al., 2020; Arentson‐Lantz, Galvan, Ellison, et al., 2019; Arentson‐Lantz, Galvan, Wacher, et al., 2019; Arentson‐Lantz et al., 2016, 2020; Björn & Per, 2004; Coker et al., 2015; Deutz et al., 2013; Dirks et al., 2014, 2016, 2019; English et al., 2016; Kawakami et al., 2001; Kitahara et al., 2003; Krainski et al., 2014; Kramer et al., 2017; Mahmassani et al., 2019; Mulder et al., 2009, 2015; Pišot et al., 2016; Ploutz‐Snyder et al., 2018; Reidy et al., 2017; Režen et al., 2014; Tanner et al., 2015; Trappe et al., 2004, 2007), 18 from an immobilization protocol (Andrushko et al., 2018; Backx et al., 2017, 2018; Christensen, Dyrberg, Aagaard, Enehjelm, et al., 2008; Christensen, Dyrberg, Aagaard, Kjaer, & Langberg, 2008; Dirks et al., 2014; Farthing et al., 2009; Hespel et al., 2001; Homma et al., 2009; Jameson et al., 2021; Kilroe et al., 2020; Mcglory et al., 2019; Oates et al., 2010; Suetta et al., 2009; Thom et al., 2001; Urso et al., 2006; Vigelsø et al., 2015; Yasuda et al., 2005) and 5 from an unloaded protocol (Campbell et al., 2013; Clark et al., 2006; de Boer et al., 2007; Mitchell et al., 2018; Tesch et al., 2004). Nine studies analyzed old subjects and 43 studies analyzed young subjects (Supplementary Table S1). Eight of the studies provided data from both the thigh and the calf and one from both the thigh and the arm. The duration of the muscle disuse protocol ranged from 5 to 84 days. The risk of bias analysis revealed that all the studies were of medium to high quality (Supplementary Table S2).

### 3.2 VO_2max_ and muscle mass loss due to disuse

Muscle disuse resulted in a significant loss of VO_2max_ (*p* < 0.0001; Table 1) and MM (*p* = 0.004; Table 1). Notably, subgroup analysis revealed that neither changes in VO_2max_ (Supplementary Figure S2) nor in MM (Supplementary Figure S3) were different between old and young subjects. The Egger test was not significant for VO_2max_ (*p* = 0.374; see also the funnel plot; Supplementary Figure S4), and the Rosenthal fail‐safe number was 256. The sensitivity analysis revealed that the study performed by Vigelsø et al. (2015) in young subjects negatively influenced the VO_2max_ trend. However, when leaving this study out of the model, the overall effect was similar (SMD: −0.973; 95% CI: −1.335 to −0.611; *I* ^2^ = 46.6%; *p* < 0.0001). Regarding MM, the Egger test was not significant (*p* = 0.272; see also the funnel plot; Supplementary Figure S5), and the Rosenthal fail‐safe number was 29. Moreover, the sensitivity analysis revealed that none of the studies influenced the results.

**TABLE 1:** Effects of muscle disuse on VO_2max_.

	VO_2max_ loss					Muscle mass loss				
Model	*N*	Effect size (95% CI)	*Τ* ^2^	*I* ^2^	*p*	*N*	Effect size (95% CI)	*Τ* ^2^	*I* ^2^	*p*
No covariates	14	−0.927 (−1.270 to −0.583)	0.191	46%	<0.0001	14	−0.337 (−0.571 to −0.104)	0	0%	0.004
Length	14	−0.017 (−0.035 to 0.0001)	0.117	34%	0.052	14	0.001 (−0.012 to 0.014)	0	0%	0.834
Initial VO_2max_	14	−0.040 (−0.066 to −0.014)	0.028	11%	0.0022	14	0.002 (−0.194 to 0.024)	0	0%	0.826
All covariates	14		0.015	6%	0.0033	14		0	0%	0.967
Length	14	−0.010 (−0.027 to −0.006)			0.203	14	0.001 (−0.013 to 0.015)			0.890
Initial VO_2max_	14	−0.033 (−0.060 to −0.007)			0.013	14	0.002 (−0.022 to 0.025)	0	0%	0.878

We reported a trend toward a negative association between the length of the intervention and VO_2max_ loss (Table 1). Baseline VO_2max_ was negatively associated with VO_2max_ loss and accounted for 85% of the total heterogeneity (Table 1). Moreover, when both covariates were introduced in the model, only the initial VO_2max_ was still negatively associated with VO_2max_ loss (Table 1). In contrast, meta‐regression analysis showed that MM decline was not potentially influenced by the length of the protocol or by the initial VO_2max_ (Table 1). Moreover, when both covariates were introduced in the model, the effect was still not significant (Table 1).

### 3.3 Strength and muscle mass loss due to muscle disuse

**FIGURE 1:**
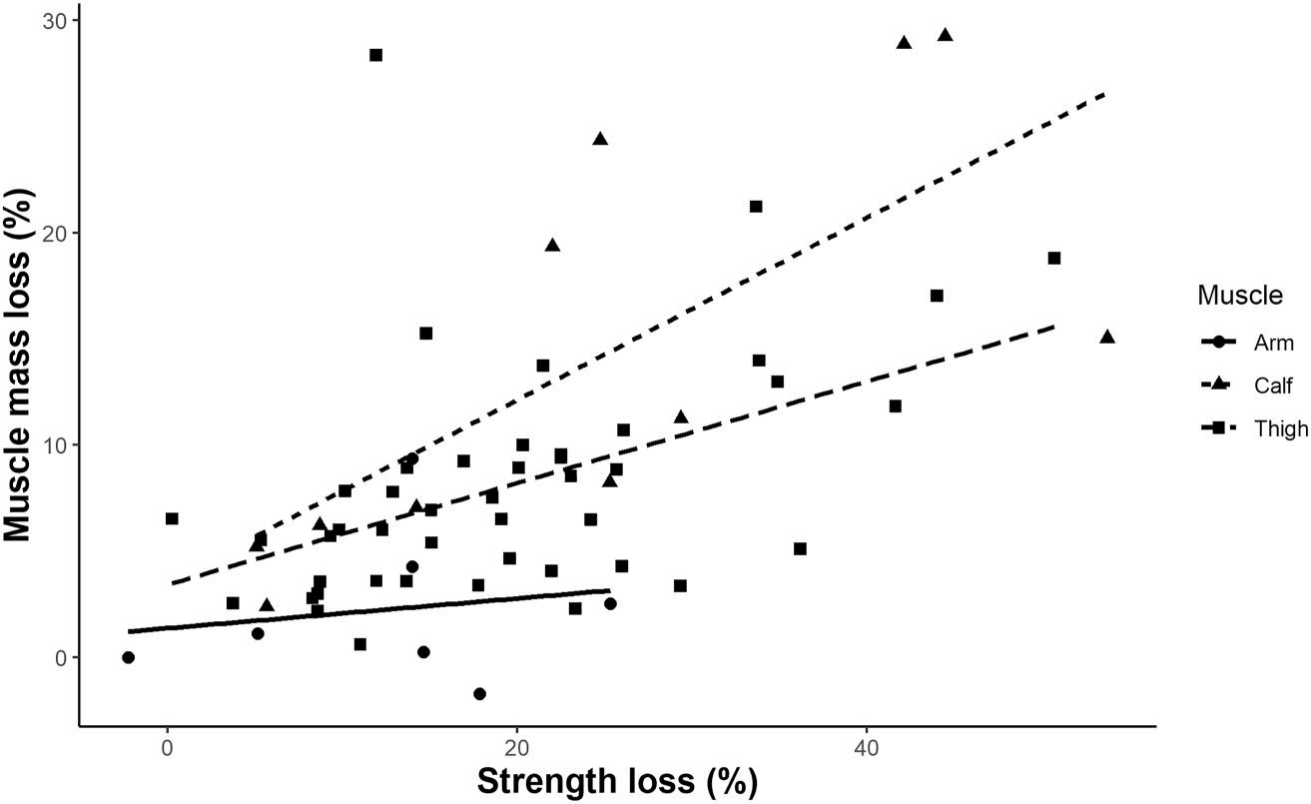
Anatomical analysis of the relationship between percent muscle strength loss and muscle mass loss.

Muscle disuse resulted in a twofold decrease in MS (SMD: −0.88; 95% CI: −1.04 to −0.73; *I* ^2^ = 43,8%; *p* = 0.0002; Supplementary Table S3) compared with MM (SMD: −0.47; 95% CI: −0.58 to −0.36; *I* ^2^ = 4.3%; *p* < 0.0001; Supplementary Table S3). Subgroup analysis showed that there were no differences between the anatomical regions analyzed for MS (*p* = 0.36) or MM (*p* = 0.20). The MS/MM effect size ratio was 1.2 for the calf, 2.2 for the thigh and 3.4 for the arms. In fact, when the percent changes in MM and MS were plotted, we found different slopes for the arm, thigh and calf (Figure 1).

MS decline was evident for young (SMD: −0.915; 95% CI: −1.088 to −0.742; *I* ^2^ = 44.9%; *p* < 0.0001; *n* = 54) and old (SMD: −0.726; 95% CI: −1.110 to −0.343; *I* ^2^ = 37.4%; *p* = 0.0002; *n* = 9). However, subgroup analysis did not reveal a difference between young and old subjects (*p* = 0.381). A similar lack of difference (*p* = 0.210) was observed for MM between young (SMD: −0.505; 95% CI: −0.630 to −0.379; *I* ^2^ = 8.8%; *p* < 0.001; *n* = 54) and old (SMD: −0.319; 95% CI: −0.580 to −0.057; *I* ^2^ = 0.0%; *p* = 0.01; *n* = 9) patients. We also found a similar MS decline (*p* = 0.825) regardless of the protocol applied, that is, bed rest (SMD: −0.935; 95% CI: −1.164 to −0.706; *I* ^2^ = 54.5%; *p* < 0.001; *n* = 33), immobilization (SMD: −0.827; 95% CI: −1.0816 to −0.5723; *I* ^2^ = 28.2%; *p* < 0.001; *n* = 23) or unloading (SMD: −0.871; 95% CI: −1.327 to −0.4149; *I* ^2^ = 0.76%; *p* < 0.001; *n* = 7).

However, subgroup analysis revealed a different MM loss between protocols (*p* = 0.031). In fact, bed rest (SMD: −0.632; 95% CI: −0.798 to −0.466; *I* ^2^ = 31.6%; *p* < 0.001; *n* = 33) showed a more powerful decline than immobilization (SMD: −0.332; 95% CI: −0.502 to −0.161; *I* ^2^ = 0.0%; *p* = 0.0001; *n* = 23) and unloading (SMD: −0.335; 95% CI: −0.644 to −0.026; *I* ^2^ = 0.0%; *p* = 0.034; *n* = 7).

For MS outcomes, we found a significant Egger test (*p* < 0.001; see also the funnel plot [Supplementary Figure S6]), and the Rosenthal fail‐safe number was 5147. Moreover, the sensitivity analysis showed one positive study (Björn & Per, 2004). When leaving this study out of the analysis, the overall effect was similar (SMD: −0.847; 95% CI: −0.992 to −0.701; *I* ^2^ = 34%; *p* < 0.0001). For MM outcomes, we found a significant Egger test (*p* = 0.005; see also the funnel plot [Supplementary Figure S7]), and the Rosenthal fail‐safe number was 1802. Moreover, the sensitivity analysis did not detect any study influencing the model. Finally, when we introduced the length of the protocol as a moderator, it was negatively associated with MS and MM loss (Supplementary Table S4).

### 3.4 Maximal knee extension strength and muscle disuse

**FIGURE 2:**
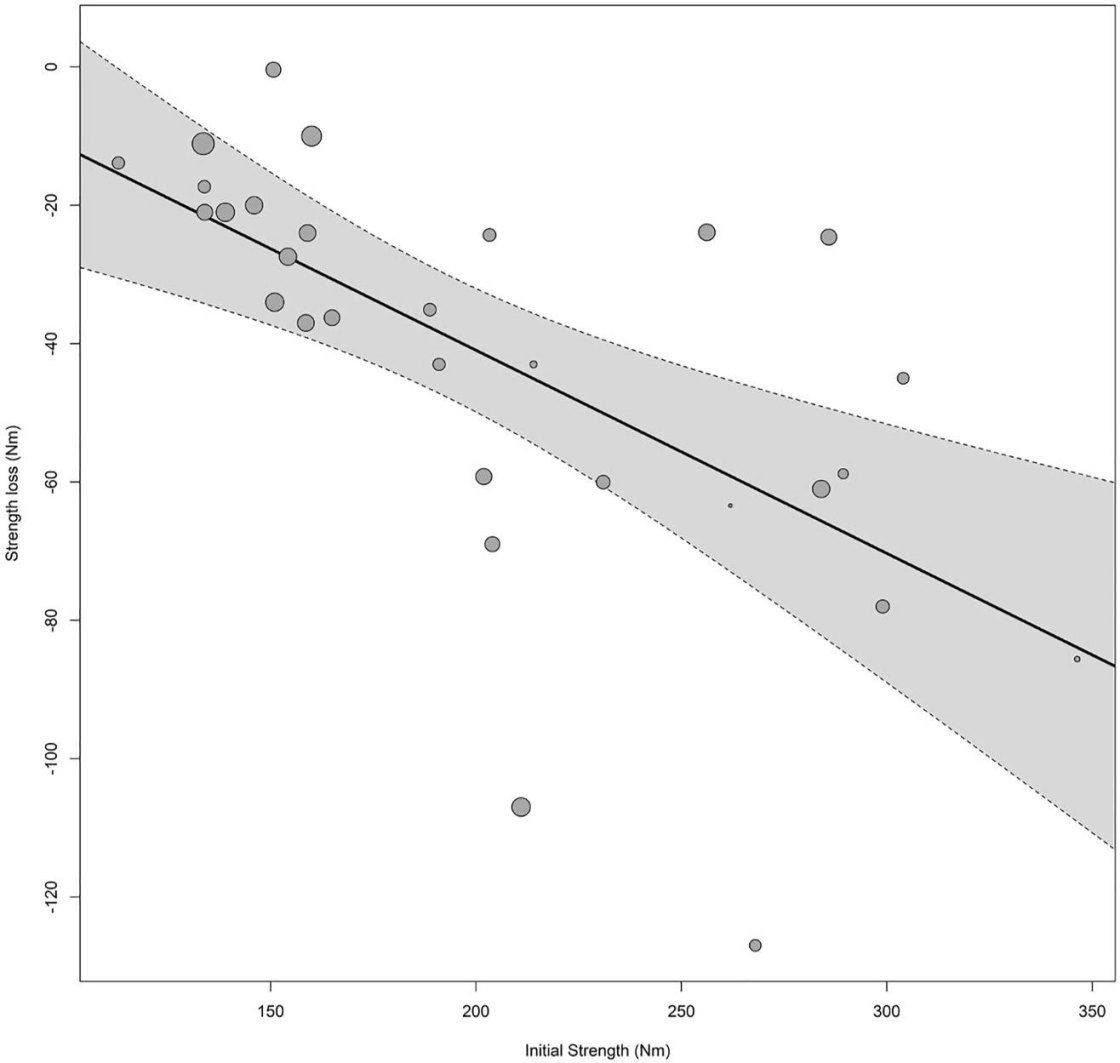
Initial strength is inversely associated with strength decline in the knee extensors.

To interrogate whether initial MS could influence muscle quality decline due to disuse, we selected studies assessing MS in the same muscle group in comparable units. A random effect meta‐analysis was performed in 30 studies (*n* = 322) reporting maximal knee extension strength (Nm) (26 analyzed isometric strength and 4 isokinetic strength). Muscle disuse resulted in a decrease in maximal isometric knee extension strength (Table 2, MD = −40 Nm). We found that the initial maximal strength of the knee extensor muscles was inversely associated with strength loss, which accounted for 50% of the heterogeneity (Figure 2). Moreover, when adjusted for the length of the protocol, initial maximal strength was still inversely associated with the change in MS (*p* = 0.003, Table 2). In fact, when both covariates were introduced in the model, 84% of the heterogeneity was explained. In contrast, initial knee extension strength was not associated with MM loss (Table 2). It should be noted that similar effects were obtained from 26 studies that assessed maximal isometric strength (Supplementary Table S5).

**TABLE 2:** Effects of muscle disuse on knee extension maximal force (Nm) and muscle loss.

	Maximal force loss (Nm)					Muscle mass loss				
Model	*N*	Effect size (95% CI)	*Τ* ^2^	*I* ^2^	*p*	*N*	Effect size (95% CI)	*Τ* ^2^	*I* ^2^	*p*
No covariates	30	−39.9 (−51.03 to −28.75)	651.6	79%	<0.0001	30	−0.413 (−0.570 to −0.256)	0	0%	<0.0001
Length univariate	30	−0.831 (−1.184 to −0.479)	201.6	53%	<0.0001	30	−0.012 (−0.020 to −0.003)	0	0%	0.008
Strength (Nm) univariate	30	−0.294 (−0.447 to −0.140)	324.8	63%	0.0002	30	−0.002 (−0.005 to 0.0008)	0	0%	0.168
Both covariates	30		105.4	36%	<0.0001	30		0	0%	0.029
Length	30	−0.669 (−995 to −0.342)			<0.0001	30	−0.011 (−0.020 to −0.002)			0.023
Strength (Nm)	30	−0.199 (−0.329 to −0.068)			0.003	30	−0.0005 (−0.003 to 0.002)			0.705

### 3.5 Quadriceps mass loss and muscle disuse

Finally, we analyzed whether initial MM could moderate muscular function decline during disuse. We identified studies assessing both maximal knee extensor strength (including squats, isometric strength, isokinetic strength and 1RM leg extension). Fourteen studies quantified Qvol (mL) (Supplementary Table S6), and 14 studies quantified quadriceps CSA (cm^2^) (Supplementary Table S7). We noticed that muscle disuse resulted in a net loss of 138 mL (*p* < 0.0001) and 4.9 cm^2^ (*p* = 0.02) for Qvol and CSA, respectively.

**FIGURE 3:**
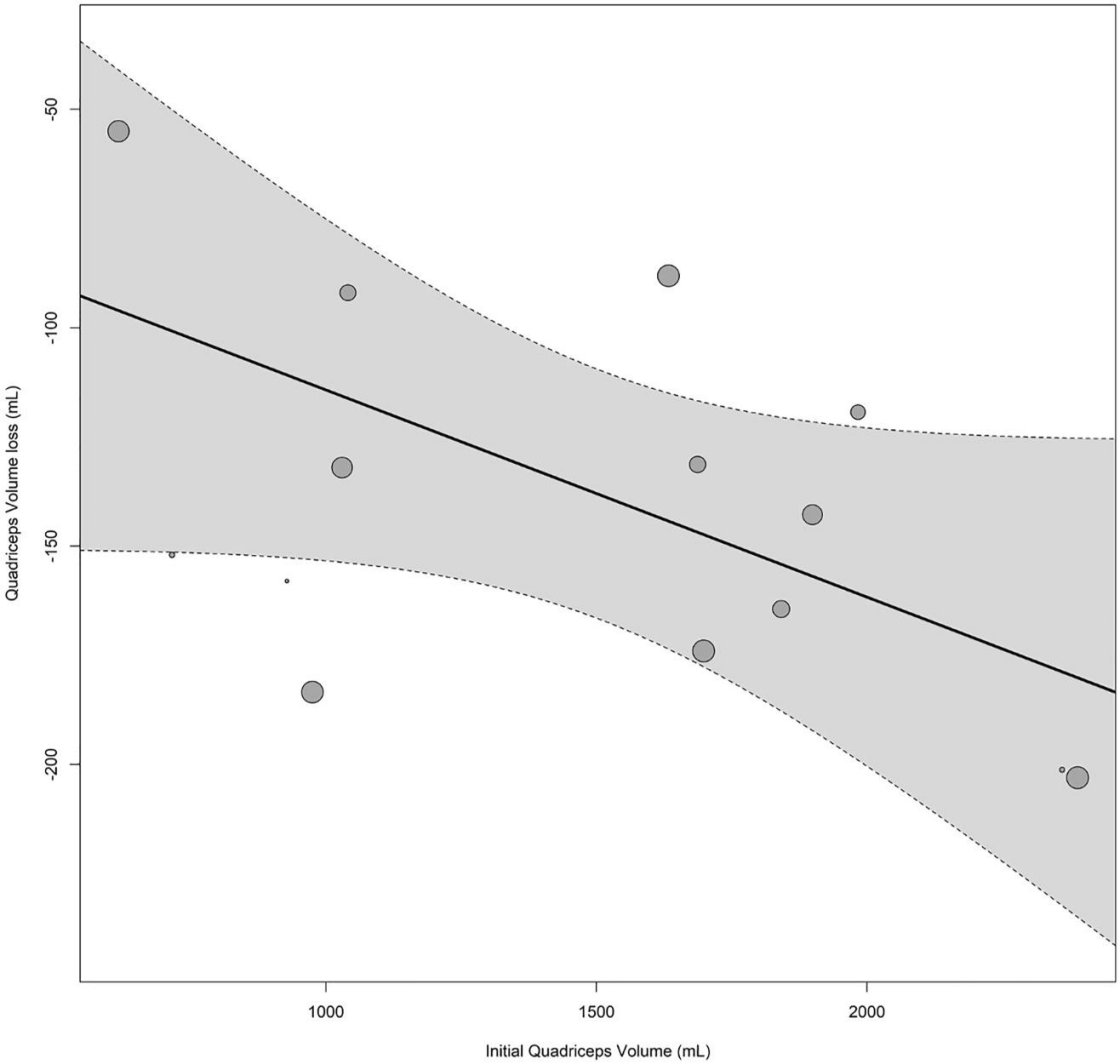
Initial quadriceps volume is inversely associated with quadriceps volume loss due to disuse.

Figure 3 shows that the initial Qvol was inversely associated with the degree of volume loss. In fact, as shown in Supplementary Table S6, when both the length of the intervention and the initial Qvol were introduced in the model, only the latter exhibited a significant inverse relationship (accounting for 44% of the heterogeneity). We found a tendency toward a lower MS decline in those subjects with higher initial Qvol (Supplementary Table S6). However, when both the length and initial muscle volume were included in the model (explaining 92.5% of the heterogeneity), only the length of the intervention inversely affected the degree of MS loss (Supplementary Table S6). Unfortunately, there were an insufficient number of studies assessing MS in comparable units to further study the relationship between the initial Qvol and MS. CSA decline was not influenced by either the initial CSA or the length of the intervention (Supplementary Table S7). Similarly, the initial CSA did not modulate MS decline due to disuse.

## 4 DISCUSSION

Muscular and cardiovascular health are fundamental to human wellbeing. Consequently, periods of muscle disuse can significantly impact a broad range of populations, from athletes to the elderly. The principal finding of our study is that functional outcomes, such as VO_2max_ and strength, decline more significantly than morphological outcomes like MM. When assessing the loss of strength and mass due to disuse, it became apparent that the decline in morphology does not always correlate with strength loss, indicating a significant neural component. This dissociation between morphological and neural components is more pronounced in the arms than in the calves. Furthermore, our meta‐regression analysis revealed that the initial level of physical fitness (i.e., VO_2max_ or strength) is inversely associated with the loss of MS and cardiorespiratory fitness.

### 4.1 Effect of muscle disuse on cardiovascular fitness

A reduction in muscle oxidative capacity (i.e., mitochondrial and microvascular function) occurs in response to muscle disuse (Abadi et al., 2009; Zuccarelli et al., 2021). Although these parameters are increased in trained subjects, it is unknown whether initial cardiorespiratory fitness could affect the degree of MM loss during disuse. Our data show that initial VO_2max_ was not associated with MM decline due to disuse. Therefore, higher cardiorespiratory fitness may not prevent disuse‐induced MM loss. However, it should be highlighted that our data are not from trained subjects, as the initial VO_2max_ was 25 and 42 mL/kg/min for old and young subjects, respectively. In addition, leg lean mass was assessed in 10 out of 14 studies using dual‐energy X‐ray absorptiometry (DXA). However, the accuracy of quantifying changes in MM over time using DXA may be lower than that of other techniques, such as magnetic resonance imaging (Tavoian et al., 2019). Thus, to clearly decipher whether the cardiovascular fitness level could alter the degree of MM decline, future studies must consider two important issues. First, researchers should carefully choose the methods for assessing MM and/or use several strategies to quantify MM. Second, subjects with a wide range of cardiovascular fitness from different ages and sexes should be included.

### 4.2 Effect of disuse on muscle strength and mass

Here, we report 63 observations of the effects of muscle disuse on MM and MS, which allowed us to assess several physiological and anatomical responses to muscle disuse. When we pooled data from studies analyzing calf, thigh, and arm muscles, we found similar responses in MM and MS between these anatomical regions. It should be noted that the proportion of type I (oxidative) fibers is ∼75% in the calf, ∼55% in the thigh and ∼35% in the arms (Saltin et al., 1977; Zinner et al., 2016). Thus, declines in muscular function are independent from the predominant phenotype of the anatomical region. In fact, several studies have reported similar atrophy rates between type I, type II (oxidative‐glycolytic) and type IIa (glycolytic) fibers in response to bed rest (Arentson‐Lantz et al., 2016; Mulder et al., 2006; Trappe et al., 2004). Our data showed a similar decline in both MS and MM between old and young subjects. Indeed, the SKM phenotype changes with age toward a higher atrophy of type II fibers (Deschenes, 2004).

It has been previously reported that calf muscles undergo greater atrophy than leg muscles in response to bed rest (Clark, 2009). In this regard, we show that calf muscles undergo twice the MM decline as thigh muscles. However, this effect was not significant, probably because we adjusted the comparison for three subgroups (thigh, arm and calf). Another discrepancy with previous studies arises when different disuse protocols are compared (Clark, 2009). We report that MM loss, but not MS loss, was higher in response to bed rest than in response to the other disuse protocols. However, the bed rest studies included in this subgroup analysis had a mean duration of 33 versus 14 days for those using immobilization. We estimated a daily decline of −0.017 for MS and −0.013 for MM. Thus, corrected MM loss would be similar between immobilization and bed rest (−0.582 vs. −0.632), and even a higher MS loss would be observed for immobilization than for bed rest (−1.15 vs. −0.935).

Importantly, we found that when corrected for length, MS loss was on average ∼2.5 times higher than MM loss (Supplementary Table S1). This is in accordance with a previous study showing that the degree of strength loss doubles in young individuals and triples in old individuals in response to immobilization (Suetta et al., 2009). This suggests a dissociation between morphological and neural adaptations in response to disuse. However, definitive studies exploring this dissociation are still needed. Based on our data, we can offer several methodological considerations to address this issue. On one hand, a greater decline in power than in maximal strength has been reported in aged muscle (Izquierdo et al., 1999). Therefore, the absence of differences in MS loss between older and younger individuals in our study could be attributed to the lack of physical tests involving explosive strength. The lack of muscle power‐related outcomes has also been reported when examining the knee extensors in response to immobilization (Preobrazenski et al., 2023). We recommend that future studies assessing muscle disuse include data on power strength. On the other hand, our results suggest that the physiological mechanisms underlying MS loss may differ between anatomical regions. For example, MM loss might contribute more significantly to MS decline in the calves than in the arms. Therefore, future research aimed at understanding the effects of neural properties on functional decline due to disuse would benefit from including a diverse sample of old and young subjects and assessing both maximal strength and power variables in both arms and legs.

### 4.3 Relationship between muscular fitness and the effects of disuse

A recent meta‐analysis inversely associated initial cardiovascular fitness with VO_2max_ decline (Ried‐Larsen et al., 2017). In addition, it has been reported that initial MM can predict MM changes following immobilization (Suetta et al., 2009). Therefore, we tested whether MS decline can be regulated by initial MS and/or MM. First, we pooled data from 30 studies assessing knee extensor muscles to show that initial MS was inversely associated with MS decline. In addition, our data indicate that initial MS cannot predict MM decline. This is in line with the notion that MM and MS differentially respond to disuse.

Next, we plotted all the studies addressing quadriceps mass in comparable units. There were 14 studies reporting CSA and 14 reporting Qvol. We found that CSA decline was weaker than the observed decline for Qvol. Moreover, when CSA was adjusted for the covariates, the effect was not significant. This finding can be explained by methodological discrepancies between studies; for instance, some studies took the CSA data from 15 cm proximal to the top of the patella (Backx et al., 2017; Dirks et al., 2016) and others from the “mid‐thigh” (Mulder et al., 2006, 2015). In addition, Yasuda et al. (2005) fixed the position at 70% from the top of the trochanter to the lateral joint space of the knee, while Mitchell et al. (2018) fixed the position at 50% of the femur length. Thus, these variations can potentially mask the effects of muscle disuse on CSA data. In contrast, when the initial Qvol was introduced in the model, we reported an inverse relationship with Qvol decline. No relationships are observed between Qvol and MS in response to disuse. This may suggest that in the thigh, MS loss is mainly induced by neural adaptations. It has, however, been proposed that the peripheral (i.e., muscular) impact on MS loss can increase along with the length of the protocol (Clark et al., 2006). Nevertheless, when we introduced the length of the study as a covariate, the initial Qvol was still not associated with MS decline.

### 4.4 Practical applications

Our findings have important applications for individuals undergoing prehabilitative exercise interventions, such as patients preparing for planned surgeries or undergoing oncological treatments. As our data shows a greater decline in strength compared to muscle size, prehabilitative exercise programs should prioritize enhancing strength over hypertrophy to limit functional decline.

In the case of unplanned muscle disuse, such as an athlete with an immobilized limb due to acute injury, rehabilitation should be individualized for the immobilized limb. For both legs and arms, the emphasis should be on maintaining MS through neural stimuli. One approach is to include contralateral resistance training (Pearce et al., 2013). In the case of calf muscles, this approach should be combined with local interventions to preserve muscle hypertrophy. However, caution should be exercised with these approaches, as studies examining different rehabilitation protocols across various anatomical regions are lacking.

### 4.5 Limitations

The main limitation of the study is that different procedures between studies can result in heterogeneity. In this regard, we performed subgroup analyses to identify differences between protocols, and we introduced the length of the interventions as a continuous moderator for the analysis. In addition, Cochran *Q* and *I* ^2^ statistics were used to control the heterogeneity between studies. Furthermore, we limited publication bias by searching for asymmetries in the funnel plot and by performing multiple sensitivity analyses to compare different meta‐analytic models under different assumptions. We also used the Rosenthal fail‐safe number to determine how many missing studies would be necessary to alter the observed results. It should also be mentioned as a limitation that we did not contact the authors from two studies with missing data.

## 5 CONCLUSIONS

In the present study, we report that in response to muscle disuse, both cardiovascular fitness and muscular strength decline to a higher extent than MM. Moreover, our data show that initial maximal strength is inversely associated with strength decline. This is relevant for athletes undergoing limb immobilization, as optimal strength is required to maintain a high‐performance level. The mechanisms underlying MS decline can differ between anatomical regions. Thus, future research needs to establish proper countermeasures depending on the immobilized limb. In this regard, no major differences were observed between bed rest, immobilization and unloading. Therefore, we suggest that the above‐mentioned mechanisms may be studied using a broad range of muscle disuse protocols.

## CONFLICT OF INTEREST STATEMENT

The authors declare that there are no competing interests. The present research does not involve human or animal research.

## Supporting information

Supplementary Material

## Data Availability

All the data are available in the manuscript or in the Supplementary Files.
