# Supplementary material for: The role of muscle disuse in muscular and cardiovascular fitness: A systematic review and meta‐regression
Source: Eur J Sport Sci. 2024 Mar 18;24(6):812–23. doi: 10.1002/ejsc.12093 (PMC11235952; doi:10.1002/ejsc.12093)

**Supplementary Files**

**Supplementary Table 1. Descriptive table.**

Table 1. Participant characteristics, resistance exercise training details and individual study outcomes

|  | ***Subject Details*** | | | |  |  |  | ***Sports Performance Assessment*** | | | | |  | ***Body Composition Outcomes*** | |  |
| --- | --- | --- | --- | --- | --- | --- | --- | --- | --- | --- | --- | --- | --- | --- | --- | --- |
| ***Author and Year*** | ***Sample*** | ***Age*** | ***Sex*** | ***BMI*** | ***Time*** | ***Procedure*** |  | ***Muscle tested*** | ***Strength*** |  | ***VO_2max_*** | |  | ***Method*** | ***Muscle*** | |
| *Akima et al., 2000* | 4 | 20 | M | 23 | 20 | Bed rest |  | Leg ext | Iso (MVC) | → | xxx | xxx |  | MRI | knee ext (Vol) | ↓ |
| *Akima et al., 2005* | 5 | 21 | M | 22 | 20 | Bed rest |  | Leg | Iso (MVC) | → | CE | ↓ |  | MRI | Thigh (Vol) | ↓ |
| *Andrushko et al., 2018* | 8 (6) | 23 | M (F) | 30 | 28 | Immob. |  | Wrist fle | Iso (MVC) | → | xxx | xxx |  | MRI | Left arm (CSA) | ↓ |
|  |  |  |  |  |  |  |  | Wrist ext | Iso (MVC) | → | xxx | xxx |  | MRI | Right arm (CSA) | → |
| *Arc-Chagnaud et al., 2020* | 10 | 34 | M | 23,7 | 60 | Bed rest |  | Knee ext | Isoc (MVC) | ↓ | xxx | xxx |  | biop | Vastus la | → |
| *Arentson-Lantz et al., 2016* | 7 (3) | 51 | M (F) | 24,8 | 14 | Bed rest |  | Knee ext | Con (MVC) | → | CE | ↓ |  | biop | Vastus la | ↓ |
|  |  |  |  |  |  |  |  |  |  |  |  |  |  | DXA | Leg (CSA) | ↓ |
| *Arentson-Lantz et al., 2019A,B, 2020* | 10 (3) | 68 | M (F) | 25,2 | 7 | Bed rest |  | Knee ext | Iso (MVC) | → | CE | → |  | DXA | Leg (CSA) | → |
|  |  |  |  |  |  |  |  | Knee ext | Iso (MVC) | → | CE | → |  | biop | Vastus la | → |
| *Backx et al., 2017, 2018* | 15 | 23 | M | 23,5 | 7 | Immob. |  | Knee ext | 1 RM | ↓ | xxx | xxx |  | biop | Quads (CSA) | ↓ |
| *Bjorn et al., 2004* | 9 | 32 | M | 24 | 90 | Bed rest |  | Leg ext | Iso (MVC) | ↓ | xxx | xxx |  | MRI | Quad (Vol) | ↓ |
|  |  |  |  |  |  |  |  | Plantar fle | Iso (MVC) | ↓ | xxx | xxx |  | MRI | Triceps S | ↓ |
| *Campbell et al., 2013* | 8 | 22 | M | xxx | 21 | Unload |  | Knee ext | Isoc (MVC) | ↓ | xxx | xxx |  | MRI | Quad (Vol) | ↓ |
| *Christensen et al., 2008A, B* | 12 (4) | 30 | M (F) | 26 | 14 | Immob. |  | Plantar fle | Iso (MVC) | ↓ | xxx | xxx |  | MRI | Gemelos (CSA) | ↓ |
|  |  |  |  |  |  |  |  | Plantar fle | Iso (MVC) | ↓ | xxx | xxx |  | MRI | Triceps S (CSA) | ↓ |
| *Clark et al., 2006* | 12 (7) | 21 | M (F) | 25 | 28 | Unload |  | Plantar fle | Iso (MVC) | ↓ | xxx | xxx |  | MRI | Calf (CSA) | → |
|  |  |  |  |  |  |  |  | Leg ext | Iso (MVC) | ↓ | |  |  |  |  |  |
| *Coker et al., 2015* | 14 (5) | 70 | M (F) | 28 | 10 | Bed rest |  | Knee ext | Iso (MVC) | ↓ | CE | ↓ |  | DXA | Leg (kg) | ↓ |
|  |  |  |  |  |  |  |  | Knee ext | Isok (1RM) | ↓ | xxx | xxx |  | DXA | Leg (kg) | ↓ |
|  |  |  |  |  |  |  |  | Knee ext | Con 60º | ↓ | xxx | xxx |  |  |  |  |
| *de Boer et al., 2007* | 9 | 19 | M | 22,5 | 23 | Unload |  | Knee ext | Iso (MVC) | ↓ | xxx | xxx |  | MRI | Quad (CSA) | ↓ |
| *Deutz et al., 2013* | 8 (1) | 67 | M (F) | 26,5 | 10 | Bed rest |  | xxx | xxx | ↓ | CE | ↓ |  | DXA | Leg (kg) | ↓ |
|  |  |  |  |  |  |  |  |  |  |  |  |  |  | DXA | Arm (kg) | ↓ |
|  |  |  |  |  |  |  |  |  |  |  |  |  |  | DXA | Total (kg) | ↓ |
| *Dirks et al., 2014* | 12 | 22 | M | 21,9 | 5 | Immob. |  | Leg ext | 1 RM (kg) | ↓ | xxx | xxx |  | MRI | Quad (CSA) | ↓ |
|  |  |  |  |  |  |  |  |  |  |  |  |  |  | Biop | Vastus la | → |
| *Dirks et al., 2016* | 10 | 23 | M | 23 | 7 | Bed rest |  | Forearm | Iso (MVC) | → | CE | → |  | DXA | Quad (CSA) | ↓ |
|  |  |  |  |  |  |  |  | Leg ext | 1 RM (kg) | → | |  |  | DXA | Quad (kg) | ↓ |
|  |  |  |  |  |  |  |  | Leg press | 1 RM (kg) | → | |  |  |  |  |  |
| *Dirks et al., 2019* | 10 | 27 | M | 23,5 | 7 | Bed rest |  | xxx | xxx |  | CE | ↓ |  | DXA | Quad (CSA) | ↓ |
|  |  |  |  |  |  |  |  |  |  |  |  |  |  | DXA | Leg (kg) | ↓ |
|  | 10 | 24 | M | 24 | 7 | Bed rest |  |  |  |  | CE | ↓ |  | DXA | Quad (CSA) | ↓ |
|  |  |  |  |  |  |  |  |  |  |  |  |  |  | DXA | Leg (kg) | ↓ |
| *English et al., 2016* | 9 (3) | 52 | M (F) | 24,7 | 14 | Bed rest |  | Leg ext | Con 60 (MVC) | ↓ | CE | ↓ |  | DXA | Leg (kg) | ↓ |
|  |  |  |  |  |  |  |  | Leg ext | Con 180 (MVC) | ↓ | |  |  |  |  |  |
| *Farthing et al., 2009* | 10 (8) | 22 | M (F) | 25 | 21 | Immob. |  | Right Arm | P Torque (Nm) | ↓ | xxx | xxx |  | Ultra | Right arm | ↓ |
|  |  |  |  |  |  |  |  | Left Arm | P Torque (Nm) | ↓ | xxx | xxx |  | Ultra | Left arm | ↓ |
| *Hespel et al., 2001* | 22 (9) | 22 | M (F) | xxx | 14 | Immob. |  | Leg ext | Iso (MVC) | ↓ | xxx | xxx |  | MRI | Quad (CSA) | ↓ |
| *Homma et al., 2009* | 15 | xxx | M | 20-29 | 21 | Immob. |  | Forearm | Iso (MVC) | → | xxx | xxx |  | MRI | Forearm (CSA) | → |
| *Jameson et al., 2021* | 11 | 20 | M | 23 | 7 | Immob. |  | Knee ext | 1 RM (kg) | ↓ | xxx | xxx |  | MRI | Quad (Vol) | ↓ |
| *Kawakami et al., 2001* | 4 | 23 | M | 23 | 20 | Bed rest |  | Knee ext | Iso (MVC) | ↓ | xxx | xxx |  | MRI | Quad (PCSA) | ↓ |
| *Kilroe et al., 2019* | 13 | 20 | M | 23,4 | 7 | Immob. |  | Leg ext | 1 RM (kg) | ↓ | xxx | xxx |  | MRI | Quad (Vol) | ↓ |
|  |  |  |  |  |  |  |  | Leg press | 1 RM (kg) | ↓ | xxx | xxx |  | MRI | Thigh (Vol) | ↓ |
| *Kitahara et al., 2003* | 6 | xxx | M | 21 | 35 | Bed rest |  | Forearm | Iso (MVC) | ↓ | xxx | xxx |  | MRI | Forearm (CSA) | → |
| *Krainski et al., 2014* | 9 (1) | 33 | M (F) | 23,3 | 35 | Bed rest |  | Knee ext | Isok (MVC) | ↓ | xxx | xxx |  | MRI | Quad (Vol) | ↓ |
|  |  |  |  |  |  |  |  | Knee fle | Isok (MVC) | → | |  |  |  |  |  |
| *Kramer et al., 2017* | 11 | 28 | M | 23 | 60 | Bed rest |  | Knee ext | Iso (MVC) | ↓ | CE | ↓ |  | DXA | Leg (kg) | → |
|  |  |  |  |  |  |  |  | Knee fle | Iso (MVC) | ↓ | |  |  |  |  |  |
| *Mahmassani et al., 2019* | 9 (7) | 23 | M (F) | 22 | 5 | Bed rest |  | Leg ext | Iso (MVC) | ↓ | xxx | xxx |  | DXA | Leg (kg) | → |
|  | 18 (7) | 68 | M (F) | 25,1 | 5 | Bed rest |  | Leg ext | Iso (MVC) | ↓ | xxx | xxx |  | DXA | Leg (kg) | ↓ |
| *McGlory et al., 2019* | 9 (9) | 22 | M (F) | 24 | 14 | Immob. |  | Knee ext | Iso (MVC) | ↓ | xxx | xxx |  | MRI | Quad (CSA) | ↓ |
|  |  |  |  |  |  |  |  |  |  |  | xxx | xxx |  | DXA | Leg (kg) | ↓ |
| *Mitchell et al., 2018* | 15 | 49 | M | 28,3 | 14 | Unload |  | Knee ext | Iso (MVC) | ↓ | xxx | xxx |  | DXA | 50% of Femur l | ↓ |
|  |  |  |  |  |  |  |  | Plantar fle | Iso (MVC) | ↓ | xxx | xxx |  | DXA | 20% of Femur l | ↓ |
|  |  |  |  |  |  |  |  |  |  |  |  |  |  | DXA | 66% of Tibial l | ↓ |
| *Mulder et al., 2006* | 9 | 33 | M | 25,2 | 56 | Bed rest |  | Knee ext | Iso (MVC) | → | xxx | xxx |  | MRI | Quad (CSA) | ↓ |
| *Mulder et al., 2009* | 9 | 33 | M | 24,6 | 60 | Bed rest |  | Knee ext | Iso (MVC) | ↓ | xxx | xxx |  | MRI | Quad (CSA) | ↓ |
|  |  |  |  |  |  |  |  | Plantar fle | Iso (MVC) | ↓ | xxx | xxx |  | MRI | left triceps S (CSA) | ↓ |
| *Mulder et al., 2015* | 10 | 30 | M | 24 | 5 | Bed rest |  | Knee ext | Iso (MVC) | ↓ | xxx | xxx |  | MRI | Knee ex (CSA) | ↓ |
|  |  |  |  |  |  |  |  | Plantar fle | Iso (MVC) | → | xxx | xxx |  | MRI | Plantar fle (CSA) | ↓ |
| *Oates et al., 2010* | 5 | 24 | M | 23,4 | 14 | Immob. |  | Knee ext | Iso (MVC) | ↓ | xxx | xxx |  | MRI | Leg (CSA) | ↓ |
|  |  |  |  |  |  |  |  |  |  |  |  |  |  | MRI | Thigh (CSA) | ↓ |
| *Pišot et al., 2016* | 7 | 23 | M | 24 | 14 | Bed rest |  | Leg ext | Iso (MVC) | → | CE | ↓ |  | MRI | Quad (Vol) | ↓ |
|  | 16 | 60 | M | 26,6 | 14 | Bed rest |  | Knee ext | Iso (MVC) | ↓ | CE | ↓ |  | MRI | Quad (Vol) | ↓ |
| *Ploutz-Snyder et al., 2018* | 8 | 37 | M | 26,3 | 70 | Bed rest |  | Leg press | Iso (MVC) | → | CE | → |  | MRI | Quad (CSA) | → |
|  |  |  |  |  |  |  |  | Leg ext | Iso (MVC) | → | |  |  | MRI | Soleus (CSA) | → |
|  |  |  |  |  |  |  |  | Plantar fle | Iso (MVC) | → | |  |  |  |  |  |
| *Reidy et al., 2017* | 10 (1) | 69 | M (F) | 25,3 | 5 | Bed rest |  | knee ext | Iso (MVC) | ↓ | xxx | xxx |  | DXA | Leg (kg) | ↓ |
| *Rezen et al., 2014* | 6 | 23 | M | 22,7 | 10 | Bed rest |  | Knee ext | Iso (MVC) | ↓ | CE | ↓ |  | DXA | Leg (kg) | ↓ |
| *Suetta et al., 2009* | 11 | 24 | M | 22 | 14 | Immob. |  | Leg ext | Iso (MVC) | ↓ | xxx | xxx |  | MRI | Quad (Vol) | ↓ |
|  |  |  |  |  |  |  |  |  |  |  |  |  |  | MRI | Quad (PCSA) | ↓ |
|  | 9 | 67 | M | 26,3 | 14 | Immob. |  | Knee ext | Iso (MVC) | ↓ | xxx | xx |  | MRI | Quad (Vol) | ↓ |
|  |  |  |  |  |  |  |  |  |  |  |  |  |  | MRI | Quad (PCSA) | ↓ |
| *Tanner et al.,2015* | 14 (7) | 22 | M (F) | 23 | 5 | Bed rest |  | Leg ext | Iso (MVC) | ↓ | xxx | xxx |  | DXA | Leg (kg) | ↓ |
|  | 9 (7) | 66 | M (F) | 25 | 5 | Bed rest |  | Leg ext | Iso (MVC) | ↓ | xxx | xxx |  | DXA | Leg (kg) | ↓ |
| *Tesch et al., 2004* | 11 | 40 | M | 26 | 35 | Unload |  | Leg ext | Iso (MVC) | ↓ | xxx | xxx |  | MRI | Quad (Vol) | ↓ |
| *Thom et al., 2001* | 8 | 23 | M | 23,5 | 10 | Immob. |  | Knee ext | 1 RM (kg) | ↓ | xxx | xxx |  | MRI | Quad (CSA) | ↓ |
| *Trappe et al., 2004* | 6 | 32 | M | 24 | 84 | Bed rest |  | Squat | Iso (MVC) | ↓ | xxx | xxx |  | MRI | Thigh (Vol) | ↓ |
|  |  |  |  |  |  |  |  | Squat | Con (N) | ↓ | xxx | xxx |  |  |  |  |
|  |  |  |  |  |  |  |  | Squat | Excentric (N) | ↓ | xxx | xxx |  |  |  |  |
| *Trappe et al., 2007* | 8 (8) | 34 | F | 21 | 60 | Bed rest |  | Squat | Iso (N) | ↓ | xxx | xxx |  | MRI | Quad (Vol) | ↓ |
|  |  |  |  |  |  |  |  | Calf press | Iso (N) | ↓ | |  |  | MRI | Triceps S (Vol) | ↓ |
| *Urso et al., 2006* | 8 | 21 | M | 26 | 14 | Immob. |  | Adductor | Sp force (N/ml^3^) | → | xxx | xxx |  | MRI | Adductor (Vol) | → |
|  | 20 | 67 | M | 28,5 | 14 | Immob. |  | Adductor | Sp force (N/ml^3^) | ↓ | xxx | xxx |  | MRI | Adductor (Vol) | ↓ |
| *Vigelsø et al., 2015* | 17 | 23 | M | 24 | 14 | Immob. |  | Leg | Iso (MVC) | ↓ | CE | → |  | DXA | Leg (kg) | ↓ |
|  |  |  |  |  |  |  |  |  |  |  |  |  |  | Biop | Leg (µm^2^) | → |
|  |  |  |  |  |  |  |  |  |  |  |  |  |  | Biop | Leg (µm^2^) | → |
|  | 15 | 68 | M | 27 | 14 | Immob. |  | Leg | Iso (MVC) | ↓ | CE | → |  | DXA | Leg (kg) | → |
|  |  |  |  |  |  |  |  |  |  |  |  |  |  | Biop | Leg (µm^2^) | → |
|  |  |  |  |  |  |  |  |  |  |  |  |  |  | Biop | Leg (µm^2^) | → |
| *Yasuda et al., 2005* | 13 | 21 | M | 24,5 | 14 | Immob. |  | Knee ext | Iso (MVC) | ↓ | xxx | xxx |  | MRI | Quad (CSA) | ↓ |
|  |  |  |  |  |  |  |  |  |  |  |  |  |  | DXA | Leg (kg) | ↓ |
|  | 14(14) | 21 | M (F) | 22,8 | 14 | Immob. |  | Knee ext | Iso (MVC) | ↓ | xxx | xxx |  | MRI | Quad (CSA) | ↓ |
|  |  |  |  |  |  |  |  |  |  |  |  |  |  | DXA | Leg (kg) | ↓ |

↓, decrease; →, no change; Immob, Immobilization; Ext, Extension; fle, flexion; Isometric, Iso, Concentric; con, Quadriceps; Quad, ergo; ergometer, Isokinetic, isok; ultra; ultrasond, Biop; Biopsies, p; peak, Sp; Specific, CE; cycle ergometer, S; surae, l; length, la; lateralis.

**Supplementary table 2:** Risk of bias

| **Criteriums according to kind of study** | | | | | | | | | | | | | | | |
| --- | --- | --- | --- | --- | --- | --- | --- | --- | --- | --- | --- | --- | --- | --- | --- |
| **Authors** | **1** | **2** | **3** | **4** | **5** | **6** | **7** | **8** | **9** | **10** | **11** | **12** | **13** | **Percentage reached** | **Quality level** |
| *Akima et al., 2000* | 1 | 1 | 1 | 1 | 1 | 1 | 1 | 1 | 1 | 1 |  |  |  | 100% | HQ |
| *Akima et al., 2005* | 1 | 1 | 1 | 1 | 1 | 1 | 0 | 1 | 1 | 1 |  |  |  | 90% | HQ |
| *Andrushko et al., 2018* | 1 | 1 | 1 | 1 | 1 | 1 | 1 | 1 | 1 | 1 |  |  |  | 100% | HQ |
| *Arc-Chagnaud et al., 2020* | 1 | 1 | 1 | 1 | 1 | 1 | 0 | 1 | 1 | 1 |  |  |  | 90% | HQ |
| *Arentson-Lantz et al., 2016* | 0 | 1 | 1 | 1 | 0 | 0 | 1 | 1 |  |  |  |  |  | 62,50% | MQ |
| *Arentson-Lantz et al., 2019A* | 1 | 0 | 1 | 1 | 1 | 1 | 1 | 1 | 1 | 1 |  |  |  | 80% | HQ |
| *Arentson-Lantz et al., 2019B* | 1 | 1 | 1 | 1 | 1 | 0 | 0 | 1 | 0 | 1 |  |  |  | 70% | MQ |
| *Arentson-Lantz et al., 2020* | 1 | 1 | 1 | 1 | 1 | 1 | 1 | 1 | 1 | 1 | 1 | 1 | 0 | 92% | HQ |
| *Backx et al., 2017* | 1 | 1 | 1 | 1 | 1 | 1 | 1 | 1 | 1 | 1 | 1 | 1 | 0 | 92% | HQ |
| *Backx et al., 2018* | 1 | 1 | 1 | 1 | 1 | 1 | 1 | 1 | 1 | 1 | 1 | 1 | 0 | 92% | HQ |
| *Bjorn et al., 2004* | 1 | 1 | 1 | 1 | 1 | 1 | 0 | 1 | 1 | 1 |  |  |  | 90% | HQ |
| *Campbell et al., 2013* | 1 | 1 | 1 | 1 | 1 | 0 | 0 | 0 | 1 | 1 |  |  |  | 70% | MQ |
| *Christensen et al., 2008A* | 1 | 1 | 1 | 0 | 1 | 0 | 1 | 1 |  |  |  |  |  | 75% | HQ |
| *Christensen et al., 2008B* | 1 | 1 | 1 | 0 | 1 | 0 | 1 | 1 |  |  |  |  |  | 75% | HQ |
| *Clark et al., 2006* | 1 | 0 | 1 | 1 | 1 | 1 | 1 | 1 | 1 | 1 |  |  |  | 90% | HQ |
| *Coker et al., 2015* | 1 | 1 | 0 | 1 | 1 | 0 | 1 | 1 |  |  |  |  |  | 75% | HQ |
| *de Boer et al., 2007* | 1 | 1 | 1 | 1 | 1 | 1 | 0 | 1 | 1 | 1 |  |  |  | 90% | HQ |
| *Deutz et al., 2013* | 0 | 1 | 1 | 1 | 1 | 1 | 0 | 1 | 1 | 1 |  |  |  | 80% | HQ |
| *Dirks et al., 2014* | 1 | 1 | 1 | 1 | 1 | 1 | 0 | 1 | 0 | 1 |  |  |  | 80% | HQ |
| *Dirks et al., 2016* | 1 | 1 | 1 | 1 | 0 | 1 | 1 |  |  |  |  |  |  | 75% | HQ |
| *Dirks et al., 2018* | 1 | 1 | 1 | 1 | 1 | 1 | 0 | 1 | 0 | 1 |  |  |  | 80% | HQ |
| *English et al., 2016* | 1 | 1 | 1 | 1 | 1 | 1 | 0 | 1 | 1 | 1 | 1 | 1 | 0 | 92% | HQ |
| *Farthing et al., 2009* | 1 | 1 | 1 | 1 | 1 | 1 | 0 | 1 | 1 | 1 |  |  |  | 90% | HQ |
| *Hespel et al., 2001* | 1 | 1 | 1 | 1 | 1 | 1 | 0 | 1 | 1 | 1 |  |  |  | 90% | HQ |
| *Homma et al., 2009* | 1 | 0 | 1 | 1 | 1 | 1 | 0 | 1 | 1 | 1 |  |  |  | 80% | HQ |
| *Jameson et al., 2021* | 1 | 0 | 1 | 1 | 1 | 1 | 0 | 1 | 1 | 1 |  |  |  | 80% | HQ |
| *Kawakami et al., 2001* | 1 | 0 | 1 | 1 | 0 | 1 | 0 | 0 | 1 | 1 |  |  |  | 60% | MQ |
| *Kilroe et al., 2019* | 1 | 1 | 0 | 1 | 1 | 0 | 1 | 1 |  |  |  |  |  | 75% | HQ |
| *Kithara et al., 2003* | 0 | 0 | 1 | 1 | 1 | 0 | 1 | 1 |  |  |  |  |  | 62% | MQ |
| *Krainski et al., 2014* | 1 | 0 | 1 | 1 | 1 | 1 | 0 | 1 | 1 | 1 |  |  |  | 80% | HQ |
| *Kramer et al., 2017* | 1 | 1 | 1 | 1 | 0 | 1 | 0 | 1 | 1 | 1 | 1 | 1 | 0 | 84% | HQ |
| *Mahmassani et al., 2019* | 0 | 1 | 1 | 1 | 1 | 1 | 1 | 1 |  |  |  |  |  | 87% | HQ |
| *McGlory et al., 2019* | 1 | 1 | 1 | 1 | 1 | 1 | 0 | 1 | 1 | 1 |  |  |  | 90% | HQ |
| *Mitchell et al., 2018* | 1 | 1 | 1 | 1 | 1 | 1 | 1 | 1 | 1 | 1 | 1 | 1 | 1 | 100% | HQ |
| *Mulder et al., 2006* | 1 | 1 | 1 | 1 | 1 | 1 | 0 | 1 | 1 | 1 |  |  |  | 90% | HQ |
| *Mulder et al., 2009* | 1 | 0 | 1 | 1 | 1 | 0 | 0 | 1 | 1 | 1 |  |  |  | 70% | MQ |
| *Mulder et al., 2015* | 1 | 1 | 1 | 1 | 1 | 1 | 0 | 1 | 0 | 1 |  |  |  | 80% | HQ |
| *Oates et al., 2010* | 1 | 0 | 1 | 1 | 1 | 0 | 0 | 1 | 1 | 1 |  |  |  | 70% | MQ |
| *Pišot et al., 2016* | 0 | 1 | 1 | 1 | 1 | 0 | 1 | 1 |  |  |  |  |  | 75% | MQ |
| *Ploutz-Snyder et al., 2018* | 0 | 1 | 0 | 1 | 1 | 1 | 1 | 1 | 1 | 1 | 1 | 1 | 1 | 85% | HQ |
| *Reidy et al., 2017* | 1 | 1 | 1 | 1 | 1 | 0 | 0 | 1 | 0 | 1 |  |  |  | 70% | MQ |
| *Rezen et al., 2014* | 1 | 1 | 1 | 1 | 1 | 1 | 0 | 1 | 1 | 1 |  |  |  | 90% | HQ |
| *Suetta et al., 2009* | 1 | 0 | 1 | 1 | 1 | 0 | 0 | 1 | 1 | 1 |  |  |  | 70% | MQ |
| *Tanner et al.,2015* | 1 | 0 | 1 | 1 | 1 | 0 | 0 | 1 | 0 | 1 |  |  |  | 60% | MQ |
| *Tesch et al., 2004* | 1 | 1 | 1 | 1 | 1 | 1 | 0 | 1 | 1 | 1 |  |  |  | 90% | HQ |
| *Thom et al., 2001* | 1 | 1 | 1 | 1 | 1 | 0 | 0 | 1 | 1 | 1 |  |  |  | 80% | HQ |
| *Trappe et al., 2004* | 1 | 1 | 1 | 1 | 1 | 0 | 0 | 1 | 1 | 1 |  |  |  | 80% | HQ |
| *Trappe et al., 2007* | 1 | 1 | 1 | 1 | 1 | 0 | 0 | 1 | 1 | 1 |  |  |  | 80% | HQ |
| *Urso et al., 2006* | 1 | 1 | 1 | 1 | 1 | 0 | 0 | 1 | 1 | 1 |  |  |  | 80% | HQ |
| *Vigelsø et al., 2015* | 1 | 1 | 1 | 1 | 1 | 1 | 0 | 1 | 1 | 1 |  |  |  | 90% | HQ |
| *Yasuda et al., 2005* | 1 | 1 | 1 | 1 | 1 | 0 | 0 | 1 | 1 | 1 |  |  |  | 80% | HQ |

HQ: high quality; MQ: medium quality.

**Supplementary Table 3**. Muscle mass and strength decline in the thigh, the arm and the

calf.

**Supplementary Table 4.** Effects of muscle disuse on strength and muscle mass.

|  | ***Maximal strength*** | | | | |  | ***Muscle mass*** | | | | |
| --- | --- | --- | --- | --- | --- | --- | --- | --- | --- | --- | --- |
| ***Model*** | ***N*** | ***Effect size (95% CI)*** | ***Τ^2^*** | ***I^2^*** | ***p*** |  | ***N*** | ***Effect size (95% CI)*** | ***Τ^2^*** | ***I^2^*** | ***p*** |
| *No covariates* | 63 | -0.882 (-1.039 to -0.725) | 0.170 | 43.8% | 0.0002 |  | 63 | -0.470 (-0.584 to -0.357) | 0.009 | 4.34% | <0.0001 |
| *Length as covariate* | 63 | -0.485 (-0.683 to – 0.287) | 0.015 | 24.5% | <0.0001 |  | 63 | -0.186 (-0.348 to 0.025) | 0 | 0% | 0.024 |
| *Length effect* | 63 | -0.017 (-0.024 to -0.011) |  |  | <0.0001 |  | 63 | -0.013 (-0.019 to 0.008) |  |  | <0.0001 |

|  | ***Strength*** | | | | |  | ***Muscle mass*** | | | | |
| --- | --- | --- | --- | --- | --- | --- | --- | --- | --- | --- | --- |
| ***Region*** | ***N*** | ***Effect size (95% CI)*** | ***Τ^2^*** | ***I^2^*** | ***p*** |  | ***N*** | ***Effect size (95% CI)*** | ***Τ^2^*** | ***I^2^*** | ***p*** |
| *All* | 63 | -0.882 (-1.039 to -0.725) | 0.170 | 44% | <0.0001 |  | 63 | -0.470 (-0.584 to -0.357) | 0.009 | 4% | <0.0001 |
| *Thigh* | 45 | -0.910 (-1.082 to -0.737) | 0.117 | 34% | <0.0001 |  | 45 | -0.459 (-0.589 to -0.328) | 0 | 0% | <0.0001 |
| *Arm* | 7 | -0.339 (-0.662 to -0.017) | 0 | 0% | 0.039 |  | 7 | -0.103 (-0.420 to 0.214) | 0 | 0% | 0.523 |
| *Calf* | 11 | -1.122 (-1.593 to -0.652) | 0.371 | 60% | < 0.0001 |  | 11 | -0.937 (-1.416 to -0.457) | 0.404 | 63% | <0.0001 |

**Supplementary Table 5.** Effects of muscle disuse on maximal isometric leg extension and muscle mass loss.

|  | ***Maximal force loss (Nm)*** | | | | |  | ***Muscle mass*** | | | | |
| --- | --- | --- | --- | --- | --- | --- | --- | --- | --- | --- | --- |
| ***Model*** | ***N*** | ***Effect size (95% CI)*** | ***Τ^2^*** | ***I^2^*** | ***p*** |  | ***N*** | ***Effect size (95% CI)*** | ***Τ^2^*** | ***I^2^*** | ***p*** |
| *No covariates* | 26 | -42.5 (-55.10 to -29.9) | 748.9 | 81.7% | <0.0001 |  | 26 | -0.380 (-0.545 to – 0.214) | 0 | 0% | <0.0001 |
| *Length* | 26 | -0.964 (-1.343 to -0.585) | 199.1 | 53.4% | <0.0001 |  | 26 | -0.015 (-0.024 to 0.005) | 0 | 0% | 0.003 |
| *Strength (Nm)* | 26 | -286 (-0.458 to -0.113) | 403 | 68.7% | 0.001 |  | 26 | -0.003 (-0.005 to 0.0001) | 0 | 0% | 0.057 |
| *Both covariates* | 26 |  | 122.5 | 39.5% | <0.0001 |  | 26 |  | 0 | 0% | 0.010 |
| *Length* | 26 | -0.795 (-1.164 to -0.425) |  |  | <0.0001 |  | 26 | -0.013 (-0.024 to -0.002) |  |  | 0.019 |
| *Strength (Nm)* | 26 | -0.160 (-0.304 to -0.017) |  |  | 0.028 |  | 26 | -0.0008 (-0.004 to 0.002) |  |  | 0.600 |

**Supplementary Table 6**. Effects of muscle disuse on quadriceps volume.

|  | ***Leg strength*** | | | | |  | ***Quadriceps volume (mL)*** | | | | |
| --- | --- | --- | --- | --- | --- | --- | --- | --- | --- | --- | --- |
| ***Model*** | ***N*** | ***Effect size (95% CI)*** | ***Τ^2^*** | ***I^2^*** | ***p*** |  | ***N*** | ***Effect size (95% CI)*** | ***Τ^2^*** | ***I^2^*** | ***p*** |
| *No covariates* | 14 | -1.116 (-1.505 to -0.727) | 0.264 | 49.9% | <0.0001 |  | 14 | -138 (-170.6 to -105.2) | 2579 | 92.1% | <0.0001 |
| *Length univariate* | 14 | -0.023 (-0.036 to -0.009) | 0.062 | 19.3% | 0.0014 |  | 14 | -0.104 (-1.559 to 1.352) | 3422 | 92.7% | 0.8889 |
| *Initial quadriceps volume univariate* | 14 | 0.0006 (-0.0000 to 0.0013) | 0.209 | 43.9% | 0.055 |  | 14 | -0.047 (-0.100 to 0.005) | 1848 | 87.9% | 0.0793 |
| *Both covariates* | 14 |  | 0.091 | 25.9% | 0.0074 |  | 14 |  | 1460 | 83.0% | 0.0350 |
| *Length* | 14 | -0.023 (-0.042 to -0.041) |  |  | 0.0171 |  | 14 | -1.109 (-2.391 to -0.173) |  |  | 0.0899 |
| *Initial Quadriceps volume* | 14 | 0.0000 (-0.0008 to 0.0007) |  |  | 0.9504 |  | 14 | -0.0785 (-0.138 to -0.019) |  |  | 0.0098 |

**Supplementary Table 7**. Effects of Muscle disuse on quadriceps cross sectional area (CSA).

|  | ***Leg strength*** | | | | |  | ***Quadriceps CSA (cm^2^)*** | | | | |
| --- | --- | --- | --- | --- | --- | --- | --- | --- | --- | --- | --- |
| ***Model*** | ***N*** | ***Effect size (95% CI)*** | ***Τ^2^*** | ***I^2^*** | ***p*** |  | ***N*** | ***Effect size (95% CI)*** | ***Τ^2^*** | ***I^2^*** | ***p*** |
| *No covariates* | 14 | -0.846 (-1.137 to -0.556) | 0.109 | 36.5% | <0.0001 |  | 14 | -4.927 (-9.081 to -0.772) | 0 | 0% | 0.020 |
| *Length univariate* | 14 | -0.007 (-0.021 to 0.007) | 0.111 | 0,37 | 0.359 |  | 14 | -0.161 (-0.578 to 0.257) | 0 | 0% | 0.450 |
| *Initial CSA* | 14 | 0.017 (-0.014 to 0.048) | 0.121 | 38.5% | 0.291 |  | 14 | 0.079 (-0.302 to 0.460) | 0 | 0% | 0.684 |
| *Both covariates* | 14 |  | 0.122 | 38.9% | 0.348 |  | 14 |  | 0 | 0% | 0.676 |
| *Length* | 14 | -0.008 (-0.022 to -0.007) |  |  | 0.318 |  | 14 | -0.168 (-0.586 to 0.256) |  |  | 0.432 |
| *Initial CSA* | 14 | 0.0182 (-0.013 to 0.049) |  |  | 0.254 |  | 14 | 0.090 (-0.292 to 0.472) |  |  | 0.644 |

**Suppl Figure 1.** PRISMA Flowchart**.**

**Identification of studies via databases and registers**

Records removed *before screening*:

Duplicate records removed (n = 413)

Records identified from*:

PUBMED n = 393

WOS n = 685

**Identification**

Records screened

(n = 633)

Records excluded**

(n = 436)

**Screening**

Reports excluded (146):

-Diseased population

-No physical fitness measurement

-No muscle mass measurement

-Animal experiments

-Countermeasures included

-Others

Reports assessed for eligibility

(n = 197)

**Elegibility**

Studies included in qualitative analysis (n= 51)

Studies included in quantitative analysis (meta-analysis)

(n = 51)

**Included**

**Supplementary Figure 2.** VO_2max_ change due to disuse.


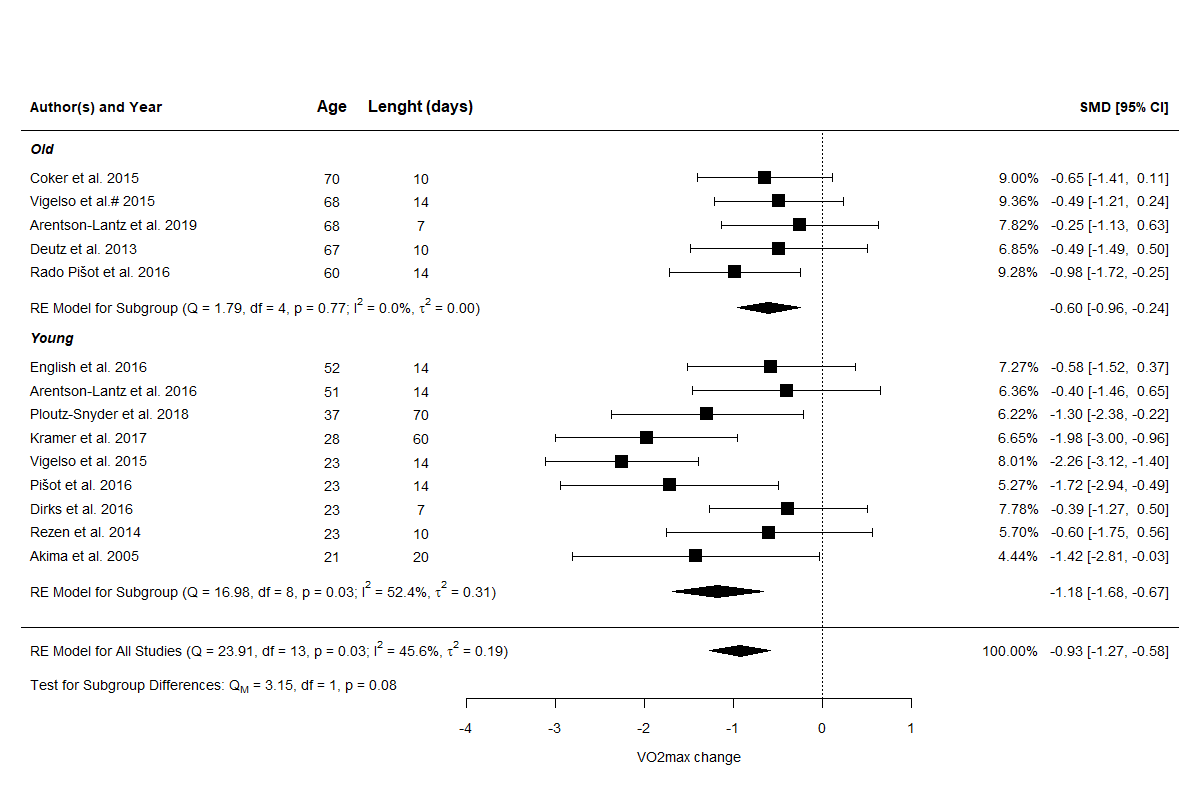


Data is shown as Standardized Mean Difference (SMD) (95% Confidence intervals). RE; random-effect model.

**Supplementary Figure 3.** Muscle mass change due to disuse in studies reporting VO_2max_.


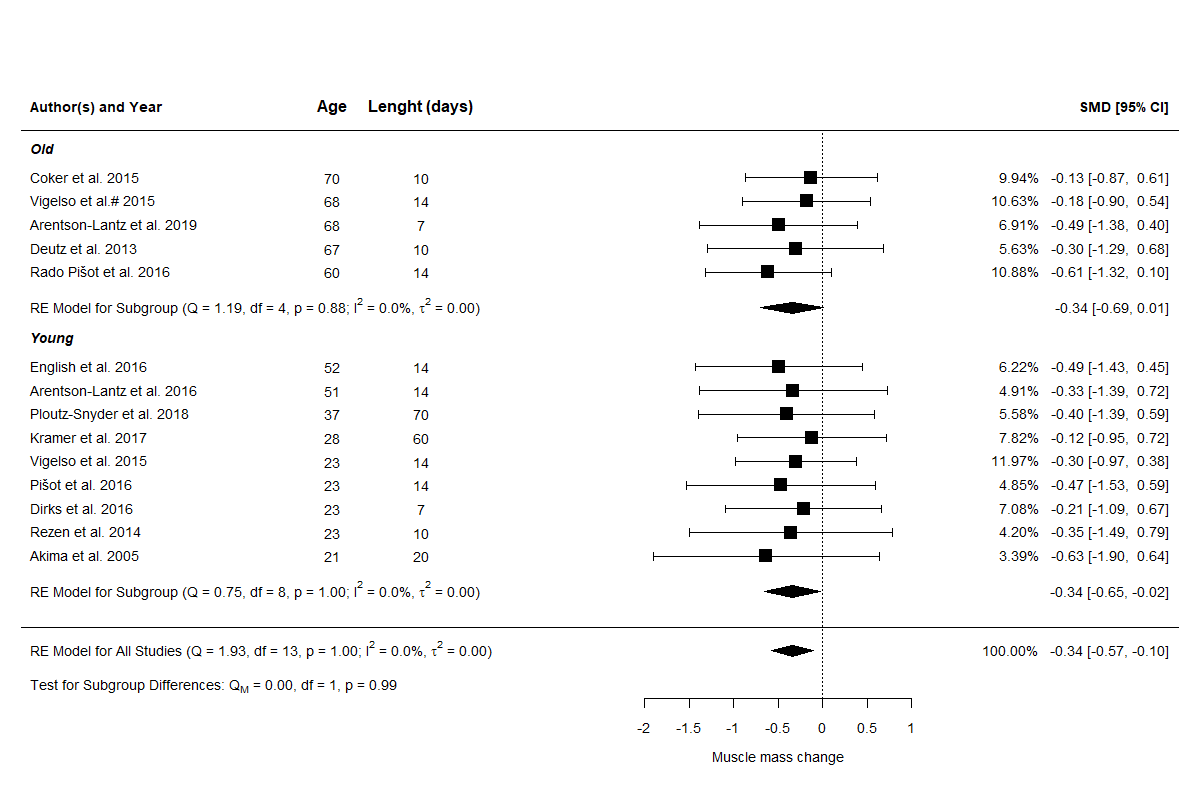


Data is shown as Standardized Mean Difference (SMD) (95% Confidence intervals). RE; random-effect model.

**Supplementary Figure 4.** Funnel plot of VO_2max_ change.


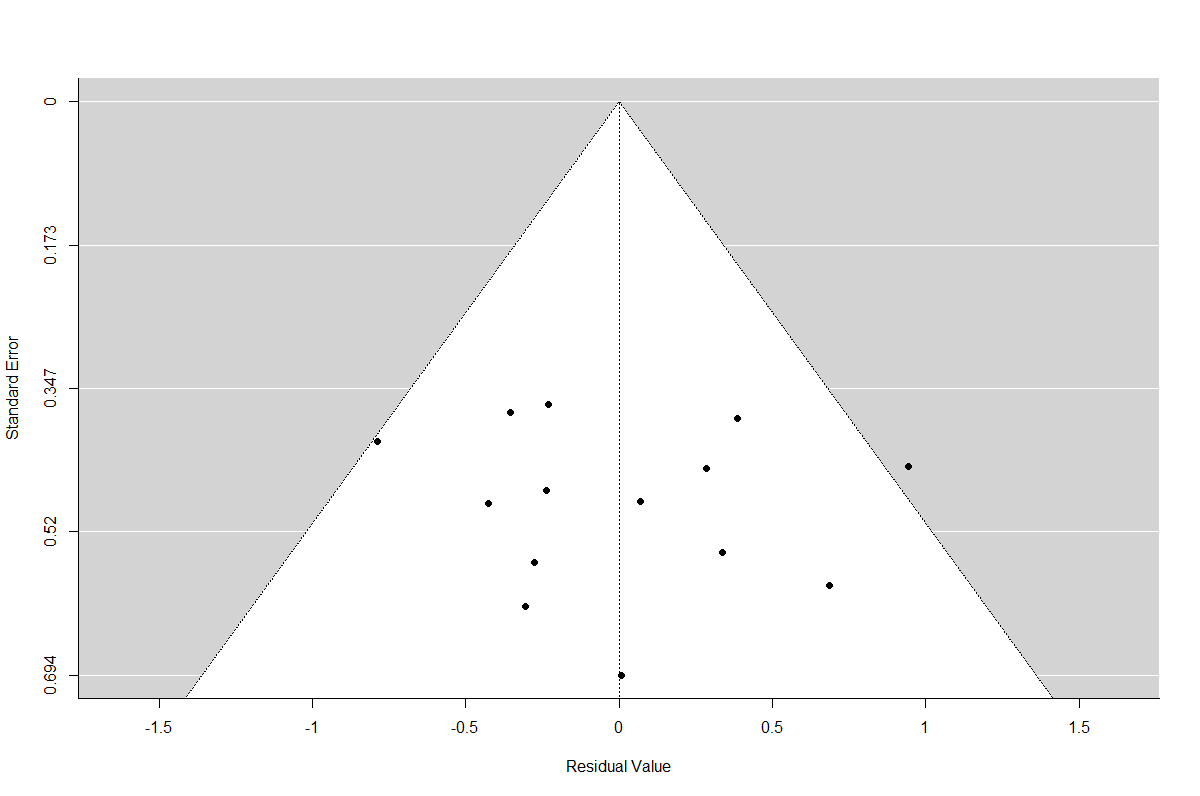


**Supplementary Figure 5.** Funnel plot of muscle mass change in studies reporting VO_2max_.
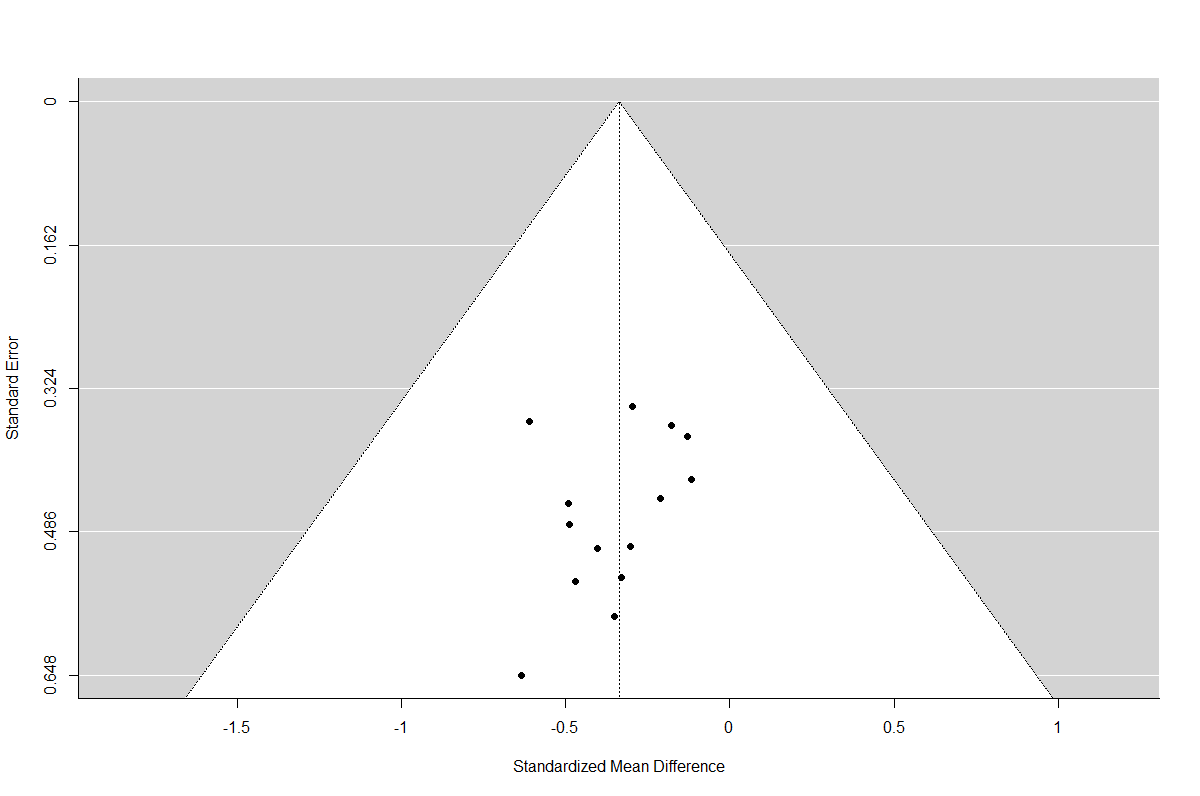


**Supplementary figure 6**. Funnel plot of strength decline


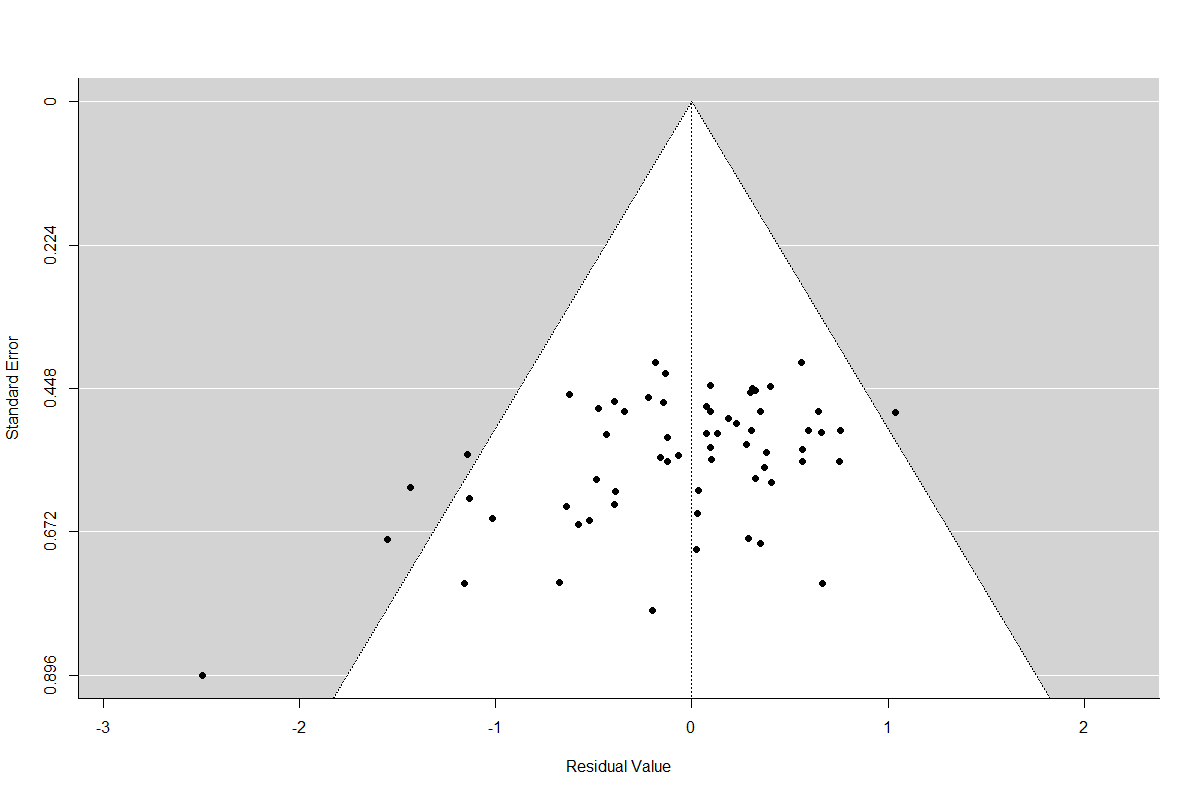


**Supplementary Figure 7**. Funnel plot of muscle mass loss.


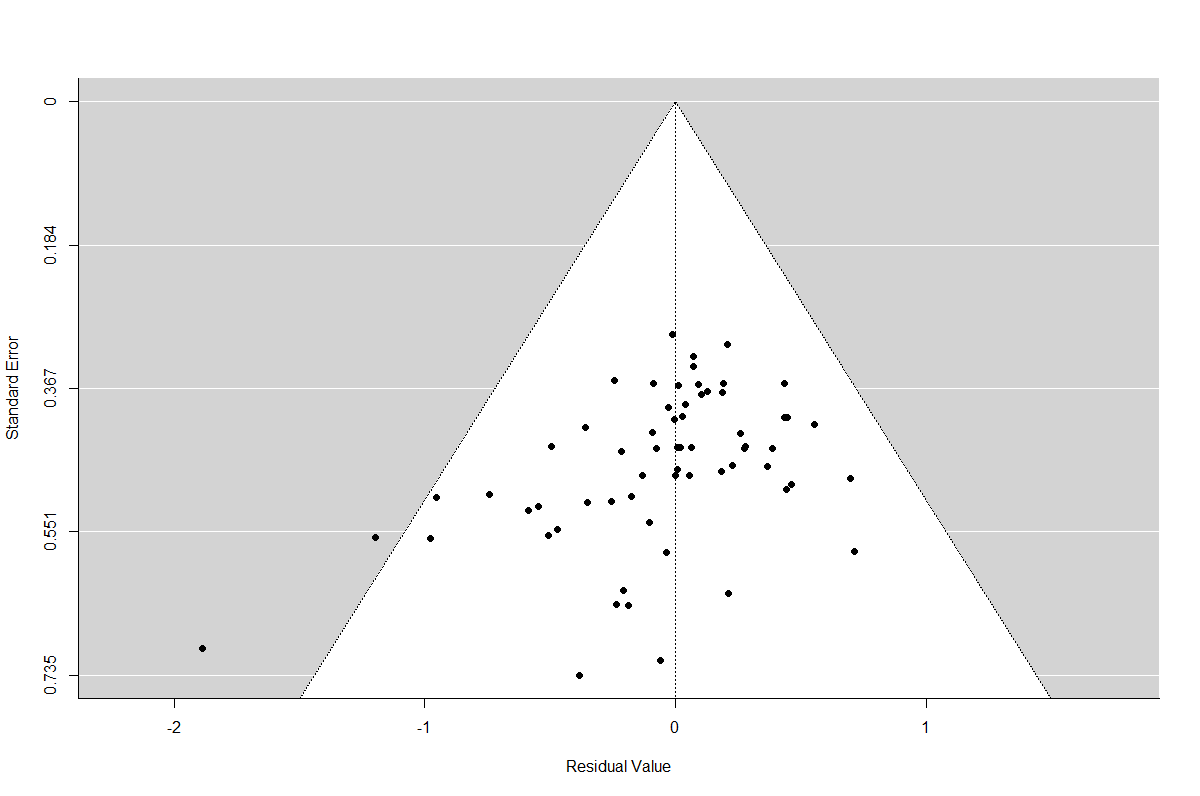

Supplement: Supplementary file 1 — Supplementary Material [file EJSC-24-812-s001.docx]
